# Pediatric Adapted Liking Survey (PALS) with Tailored Nutrition Education Messages: Application to a Middle School Setting

**DOI:** 10.3390/foods10030579

**Published:** 2021-03-10

**Authors:** Rachel Hildrey, Heidi Karner, Jessica Serrao, Carolyn A. Lin, Ellen Shanley, Valerie B. Duffy

**Affiliations:** 1 Department of Allied Health Sciences, University of CT, Storrs, CT 06269-1101, USA; rachel.hildrey@uconn.edu (R.H.); heidi.karner@uconn.edu (H.K.); jessica.serrao@uconn.edu (J.S.); ellen.shanley@uconn.edu (E.S.); 2 Communications Department, University of CT, Storrs, CT 06269-1101, USA; carolyn.lin@uconn.edu

**Keywords:** nutrition, diet, physical activity, school-based, obesity prevention, eHealth, tailored interventions, sleep, food insecurity, children, adolescents

## Abstract

We tested the feasibility of a school-based, liking-based behavioral screener (Pediatric Adapted Liking Survey (PALS)) and message program to motivate healthy diet and activity behaviors. Students, recruited from middle- (*n* = 195) or low-income (*n* = 310) schools, online-reported: likes/dislikes of foods/beverages and physical/sedentary activities, scored into healthy behavior indexes (HBI); perceived food insecurity; and sleep indicators. Students received tailored motivating or reinforcing messages (aligned with behavior change theories) and indicated their willingness to improve target behaviors as well as program feasibility (acceptability; usefulness). Although HBIs averaged lower in the lower versus middle-income school, frequencies of food insecurity were similar (39–44% of students). Students in both schools reported sleep concerns (middle-income school—43% reported insufficient hours of sleep/night; low-income school—55% reported excessive daytime sleepiness). Students across both schools confirmed the PALS acceptability (>85% agreement to answering questions quickly and completion without help) and usefulness (≥73% agreed PALS got them thinking about their behaviors) as well as the tailored message acceptability (≥73% reported the messages as helpful; learning new information; wanting to receive more messages) and usefulness (73% reported “liking” to try one behavioral improvement). Neither message type nor response varied significantly by food insecurity or sleep measures. Thus, this program feasibly delivered students acceptable and useful messages to motivate healthier behaviors and identified areas for school-wide health promotion.

## 1. Introduction

Although adults [1] and children [2] report that “taste” is a primary driver of food choice, it is the liking of the taste of food that drives what is chosen and ultimately consumed. Reported liking of foods and beverages is part of the broad taxonomy of dietary behaviors [3] that can serve as a proxy for what is consumed. Market survey data showed that the correlation between liking and ratings of consumption can range from 0.52 to 0.67 [4]. However, liking is much easier to recall and takes much less skill and time to complete than is recalling what was consumed and translating this into frequency categories and/or records of dietary intake, especially for children. We have found that responses to the Pediatric Adapted Liking Survey (PALS), reported by parents on their preschoolers’ liking for foods and beverages, can be formed into an index of diet quality that correlates with skin carotenoid status [5]. Older children can complete the PALS by themselves to produce reliable food groups [6,7] and, when combined with physical activities and screen time, formed a valid and reliable healthy behavior index [8]. Thus, the liking survey allows feasible phenotyping of the child’s diet and physical activity behaviors. It is of interest here if reported liking in children could be leveraged for promoting healthier behaviors at the individual and community levels for the prevention of chronic diseases.

Overweight and obesity is a significant disease in children. The 2018–2019 National Survey of Children’s Health revealed a 15.5% prevalence of obesity among U.S. children ages 10–17 years [9]. Highest rates of obesity are seen among Hispanic and non-Hispanic black children [10], due to complex interactions among families who have lower education and income [11] as well as food insecurity [12]. To help prevent obesity among economically disadvantaged children, school-based activities need to be deemed acceptable to diverse children, without the appearance of being discriminant based on child or school characteristics. By implementing a healthful school meal program, health education, physical activity, parent engagement, community interactions, and school wellness policies, schools are uniquely positioned to provide opportunities for all students to practice enjoyable healthy eating and physical activity [13]. Even so, school-based health promotion programs were found only to have small effects on obesity prevention efforts through improving dietary habits and physical activity [14]. These types of programs did, however, generate significant impacts on enjoyable physical activity and helped identify the need to improve school-based dietary interventions [15]. 

Precision nutrition aims to tailor interventions to individuals, recognizing a multitude of factors that influence an individual’s phenotype and variation in response to interventions [16]. For this reason, there is a need for designing school-based interventions that recognize individual phenotypes within the broader school-wide activities [17]. The landmark, multi-country randomized controlled trial, Food4Me, suggested that technology could deliver interventions and that personalizing recommendations to reported dietary intake was effective [18]. Based on this evidence, the question is whether technology could be leveraged in a school setting with a liking-based phenotype to tailor a school-based intervention to children. In a clinical setting, we have shown that messages can be tailored to children’s liking responses to deliver tailored health promotion messages [7]. In particular, children, ages 5 to 17 years, reported that the PALS was easy to complete and got them to think about their behaviors; they also indicated that the tailored messages were informative and helpful, and that they were willing to try healthier behaviors in response to their message exposure. 

In essence, surveying food liking can offer a useful proxy for measuring consumption in adolescents for sensory phenotyping [19]. Health information also can be tailored to an individual’s readiness to change as explained in the transtheoretical model (TTM) [20]. The original TTM has five stages and asked individuals to report where they are in the behavior change process—pre-contemplation, contemplation, preparation, action, maintenance. For children and evidenced-aligned [21], a two-stage approach (pre-action, action/maintenance) from the TTM to the liking survey has been found in previous research to be effective in delivering motivating messages to children who reported liking less healthy foods and targeted reinforcing messages to children who reported liking healthy foods [7]. 

This study aimed to determine the feasibility of using the online PALS with message program in a school setting—including tailoring the messages to the child’s liking phenotype, providing direction for school-wide message campaigns, and expanding the ability to reach all children—including those of socio-economic disparity. The aim was to assess feasibility, operationalized as the acceptability and usefulness of the program to the children [22], as well as toward the goal of motivating healthier behaviors for obesity prevention and providing usable information for school-based health promotion interventions. Through collaboration with school stakeholders, the online PALS was adapted from reaching children and families in a clinical setting [7] to reaching children in a school setting, incorporating questions with a focus on food insecurity, and school meal acceptance. Stakeholders in both school settings also reported interest in evaluating poor sleep habits since most middle schoolers report not meeting recommended hours of sleep [23] and insufficient sleep associates with lower academic achievement [24], less healthy diet- and physical activity-behaviors, as well as increased risk of obesity [25]. Thus, specific study aims included: assessing the diet and physical activity behavioral needs in each school; examining the acceptance and usefulness of the PALS; describing the tailored messages that children received and their willingness to work on target healthy behaviors; examining the message and their usefulness; and determining if the tailored messages or responses varied by perceived food insecurity or sleep measures. Because the message program was designed to promote healthy behaviors for obesity prevention and to reach high-need children, the acceptability and usefulness of the PALS and tailored messages was evaluated across the schools and by food insecurity and sleep status. 

## 2. Materials and Methods 

### 2.1. Participants

This online survey study with brief tailored messaging involved students in two suburban middle schools in Connecticut, USA. In the fall of 2018, 7th graders were recruited from School A, in which 39% of the student body qualified for federally funded free or reduced cost school meals. In the fall of 2019, 6th, 7th, and 8th graders were recruited from School B, in which 65% qualified for free or reduced cost meals. The schools selected were based on shared goals between the school stakeholders and research team to improve the school meal program and promote healthy behaviors for obesity prevention of their students. The study was conducted in accordance with the Declaration of Helsinki, and the protocol was approved by the Internal Review Board of the University of Connecticut (Project identification H18–032). Parents and guardians were informed about the study through Parental Notification Forms sent home. Any parents and/or students who did not wish to participate were instructed to submit a Notification of Refusal Form to the school. All procedures took place online. Neither the school staff, the research team, nor student peers were able to match the children’s identity from their responses or the messages they received. 

The survey was administered to all students during school hours to ensure every student in the described grade levels had the opportunity to take part. Students assented to participate via the face page of the online survey. Students who did not wish to or assent to participate were to receive a supplemental online assignment. All students, who received a study invitation, chose to participate in the study. Absent students completed the survey the next day they were back in school. Since the survey was only conducted once per school, there was no possibility for students to drop out. However, students could quit the survey at any time. Students who did not respond to all the survey questions were removed from the study sample (<5%). 

### 2.2. Procedure

The ADAPT-ITT model, modified, provided a framework to adapt the clinically based survey and tailored message program to a school setting (Table 1) [26]. The research team worked with school stakeholders to identify their needs and adapt the program to a school setting, including measuring acceptance of the school meal program, food insecurity, and sleep. Students completed the online survey, including PALS with messages via a secure Qualtrics platform (Provo, UT, USA) during a single session. For School A, students completed the program during science class on school-provided Chromebooks; for School B, students completed the program during science and social studies classes on school-provided desktop computers. 

The program began with having the students review an informational sheet on which they provided or declined to assent to participate in the study. Following this, students were asked to respond to the Pediatric-Adapted Liking Survey (PALS), including liking of school breakfast and school lunch; demographic information; perceived food security; indicators of sleep status; PALS usefulness and acceptance; health messages tailored to PALS responses (using an algorithm embedded in Qualtrics); and message usefulness and acceptance (as described below). 

### 2.3. Characteristics of the Student

Students across schools were asked to report their age, gender (male/female/other), race, ethnicity, and how they were feeling. Responses to “How do you feel today?” were reported using a facial hedonic scale with seven faces, without word labels, and a slider that allowed a rating from 1–7 (1 = face with the biggest smile; 7 = face with the biggest frown). 

The “sleep” measure administered was different between the two schools as described in Table 1 from applying the ADAPT-ITT Model [26]. School A students were asked what time they usually went to bed and then what time they usually woke up to derive usual hours of sleep per night. Sleep hours were evaluated for sufficiency using the National Sleep Foundation guidelines, which state that children ages 6–11 years should get 10–11 hours of sleep per night and children ages 12–14 years should get 8.5–9.5 hours of sleep per night [27]. 

The method in which sleep was assessed was modified for School B because the stated desire of the stakeholders to measure the students’ functional responses to lack of sleep. Thus, School B students reported daytime sleepiness using an adaptation of the 8-item, validated Pediatric Daytime Sleepiness Scale (PDSS) [24]. In the present study, students reported the daytime sleepiness items on a scale identified by time increments (everyday = 4, 2–3 times per week = 3, once per week = 2, 2–3 times per month = 1, never = 0) instead of Likert-scale categories (always = 4, frequently = 3, sometimes = 2, seldom = 1, never = 0) to improve comparison of sleepiness across students [28]. The responses to the 8-items were summed into a single score (possible range from 0 to 32). Scores on the PDSS ≥15 were categorized as insufficient sleep, having been correlated with poorer academic and health outcomes in adolescents [29]. 

Since asking children directly about food insecurity may improve their understanding of the impact of their diet on health outcomes [30], students reported “never,” “sometimes,” or “often” to the following three questions: “I was hungry but didn’t eat enough because there wasn’t enough food at home”; “I felt worried that our food at home would run out before we could get more”; and “I ate less than I wanted because there wasn’t enough food at home.” Students who responded “sometimes” or “often” to one of three questions about food insecurity were considered to be food insecure. Only students who responded “never” to all three questions were considered to be food secure. The online survey method may produce more honest responses from students because it improves their perceived anonymity and stigma by utilizing a digital modality that is familiar to them [31,32].

### 2.4. Behavioral Screening and Tailored Message Program 

The PALS provides a screener for dietary intake and physical activities that has established test-retest reliability [6] and has been content and criterion validated in children [8]. Presently, students were asked to report online their liking/disliking of 1 practice item (fun parks) and 33 measurement items, including a randomized set of foods and beverages, physical activities, sedentary behaviors, school breakfast, and school lunch. The student saw each item as a picture with a label to the left and a facial hedonic scale with seven labeled faces to the right, which had a slider to allow a continuous rating from ±100. The middle point of the labeled faces had the following values: ±88 “Love it”/” Hate it,” ±59 “Really Like”/” Dislike it,” ±30 “Like it”/” Dislike it,” and ±10 “It’s okay.” The individual measurement items for foods and beverages, physical activities, and sedentary behaviors were conceptually grouped into eight food groups and two activity groups—as the basis for triggering the tailored messages—and to allow for comparison across the schools. 

After the students completed the PALS, their responses automatically triggered health promotion messages that were delivered to them online, similar to the method used in the study conducted in a clinical setting [7]. These messages were intended to motivate healthier behaviors or reinforce current healthy behaviors through fun images and attention-grabbing words, while avoiding disparaging messages on weight or dieting and feelings of weight stigma [33]. 

Following the elaboration likelihood model of attitude change [34], we aimed to deliver two to three messages (motivation and/or reinforcing) to each child that were tailored to the child’s average liking/disliking responses for each food or activity group. Aligned with a two-stage transtheoretical model of behavior change [35] and criteria derived from a separate group of children (*n* = 525) in a past study [36], one or multiple of the eight motivational messages were delivered to children who reported a high liking for the “moderation” groups or low liking for the “adequacy” groups. Of these motivational messages, four of the messages aimed to reduce less healthy foods by replacing them with healthier options for salty snacks, sugar sweetened beverages, sweets, and sedentary behaviors (referred to in the results as “replace healthier”) and four of the messages aimed to add healthier options to the diet, including vegetables, whole grains, dairy products, and fruits (referred to as “add healthier”). The motivational messages in the present study are a slight rewording of those reported for use in a clinical setting [7]. 

Students also could receive one or multiple of the five reinforcing messages if they reported liking healthier food groups at a high enough level to trigger a message that highlights dairy products, fruits, vegetables, whole grains, and physical activity. For example, if the student reported above “really liking” the group of vegetables, they would receive the reinforcing message, “Keep crunching on veggies! The more you eat the better—they’re packed with vitamins and fiber.” The number of motivational and reinforcing messages each child received was calculated. 

For analysis, the children were categorized as receiving only motivational messages, only reinforcing messages, and receiving both motivational and reinforcing messages. 

Some students did not meet the criteria to receive a tailored message, instead receiving only two generic messages that all students also received. The generic messages encouraged drinking water instead of soda and avoiding food waste at school. In order to compare reported behaviors across the schools and to compare with food insecurity and sleep indices, a Healthy Behavior Index (HBI) was constructed for each student following the methodology described in our previous study [8]. For constructing the HBI, each PALS group (food/beverages, physical activity, and sedentary behaviors) was theoretically weighted following the U.S. Dietary Guidelines 2015–2020 [37]. That is, each group was weighted through multiplying by a positive number for adequacy groups (vegetables*3, fruits*2, protein*2, fiber*2, dairy*2, physical activity*2) and a negative number for moderation groups (sweets* −3, sugary beverages* −3, salty* −2, screen time* −3). The weighted groups were then averaged into an overall HBI for each student.

### 2.5. Feasibility Measures (Acceptability and Usefulness)

Before receiving the health promotion messages, children were asked to report on their level of agreement/disagreement about the PALS. Specifically, they were asked about the PALS acceptability (“I could answer the questions quickly without help;” “I could fix my mistakes easily and quickly”) and usefulness (“The questions made me think about what I eat and what I do”), whose measures were based on adapting a single item each from the three usability constructs of the Usefulness, Satisfaction, and Ease (USE) Questionnaire [38]. These questions were reported on a scale similar to that of reporting level of liking/disliking, except with seven labels ranged from “strongly agree” (1) to *“*strongly disagree” (7). After receiving all messages and with the same scale, the students were asked their agreement/disagreement with the message acceptability (“I learned new information about food and nutrition from these messages;” “The messages I received were helpful”) and usefulness (“I would like to receive more messages like these in the future”). 

Immediately after receiving each health promotion message (tailored or generic), the students rated their level of liking/disliking on trying the target behaviors suggested in the messages. The focus of the present study was students’ willingness to try a healthier behavior in response to a motivational message. For example, "Please think about the vegetable message you just received. How much would you like to eat more vegetables?” Students responded to this question using the same scale used for the PALS responses described above. 

### 2.6. Data Analysis 

Data were analyzed using Microsoft Excel (version 15.13.1) and SPSS (version 25.0) with significance criterion of *p* ≤ 0.05. The datasets from each school were combined with labels indicating the school membership of each student; there was consistency across the schools for the PALS survey and tailored message program, as well as the acceptability, usability, food security, and demographic variables. The sleep variable differed and was retained as separate variables and categorized as sufficient/insufficient for the study sample description.

Descriptive statistics presented demographic and health data, PALS responses, message number, and tailored message type, as well as acceptability and usability of the PALS and tailored messages. Mean differences are reported with the standard error of the mean. A Kolmogorov–Smirnov test was used to compare the distribution of age and school meal acceptability across schools. Differences in PALS responses, healthy behavior index scores, message acceptance and usability, and willingness to work on target behaviors were compared between the schools via child demographics and characteristics (food insecurity status and sleep). These comparisons were assessed by non-parametric statistics (*X*^2^ for categorical and Spearman rho statistic for continuous variables) and analysis of covariance (ANCOVA)—controlling for age, gender, and the liking of fun parks to control for scale usage [28]—with Levene’s test for homogeneity of variances across levels of the independent variables. A Kolmogorov–Smirnov test further differentiated between the healthy behavior index scores from the two schools. Parametric statistics were used to assess the psychometric properties of the HBI, including Cronbach’s alpha for the internal reliability of the index and principal component analysis to examine the factor structure of the index. Standard linear regression was applied to assess the unique relationship of PDSS on the healthy behavior index scores, while controlling for the effects of the reported liking of fun parks, age, and gender. 

## 3. Results

### 3.1. Descriptive Results

Table 2 shows the characteristics of the 505 middle schoolers in both schools who completed the program. The distribution of age in School B was statistically different from School A—with greater numbers of students above and below the mean of 12.0 ± 0.03 years (Kolmogorov–Smirnov *Z* = 2.855, *p* < 0.01)—whereas the distribution by gender was balanced. School B had more diversity in race/ethnicity than School A, with significant differences across the categories of White, Black/African American, and Hispanic/Latino (*X*^2^ (2) = 58.94, <0.01). Approximately 41% of the students reported food insecurity. Despite more School B families having lower income status to qualify for federal school meal assistance than School A, the frequency of food insecurity did not differ significantly between the two schools (*X*^2^ (1) = 0.99, 0.32). 

### 3.2. Description of the PALS Responses

**Figure 1:**
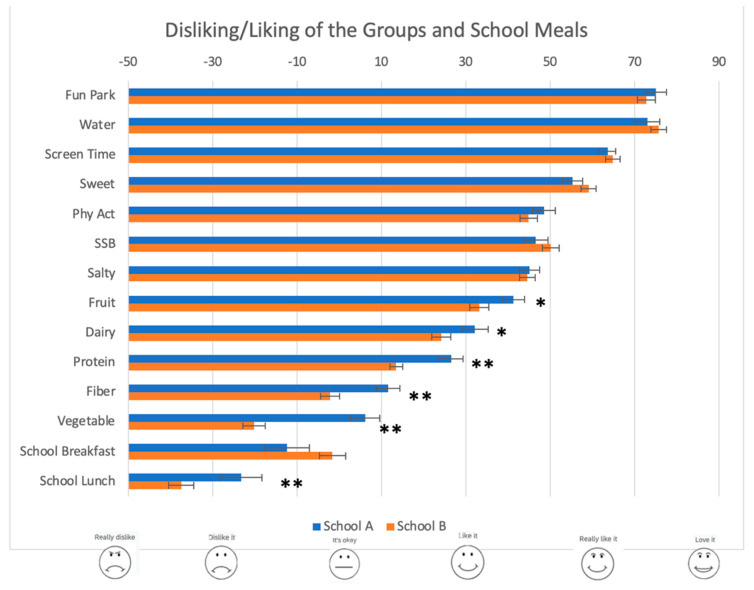
Students’ average reported liking of foods and behaviors at both schools. The *,** indicates *p* < 0.05; School B had significantly lower liking of healthier groups (protein, fiber, and vegetables).

Both schools showed good variability in liking/disliking ratings across the food and activity groups (Figure 1), with the level of liking of fun parks and water averaging most liked and not differing significantly across the two schools (*p* > 0.25). The order of ranking of the groups from highest to lowest was nearly equivalent. The healthier food groups were ranked as least liked in both schools, yet students in School A reported significantly greater average liking for the healthier food groups than students in School B (*p* < 0.05). 

**Figure 2:**
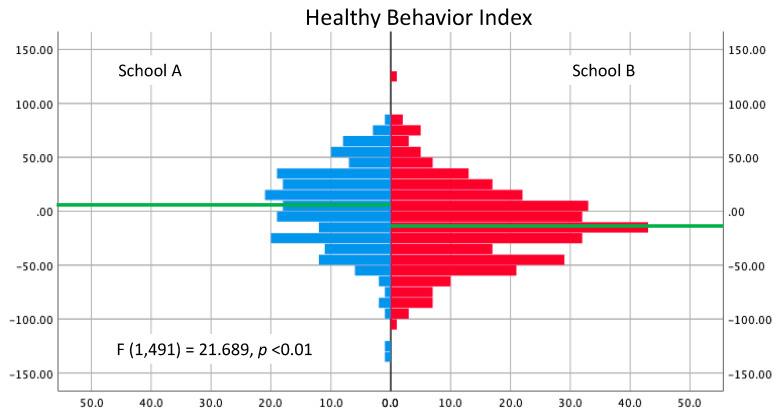
Distribution of the healthy behavior index (HBI) scores for School A and School B, with the horizontal green line approximating the mean difference in each school.

The healthy behavior index scores across both schools showed good variability and normal distribution (Kolmogorov–Smirnov, *p* = 0.2) (Figure 2). The HBI showed good psychometric properties, including an internal reliability approaching acceptable (Cronbach’s alpha = 0.66) and principal component analysis showing three factors that explained 58% of the variability across the sample (27% less healthy groups, 21% healthy groups, 10% physical activities). School B had a distribution leaning toward significantly lower healthy behavior index (HBI) scores than that for School A (Kolmogorov–Smirnov *Z* = 2.427, *p* < 0.01). Similarly, the average HBI were significantly lower in School B versus School A (−13.08 ± 2.12 versus 3.07 ± 2.74) in a one-way ANCOVA, controlling for gender, age, and liking of fun parks (F (1, 491) = 21.689, *p* < 0.01).

Across both schools, the school lunch program averaged least liked than all food or activity groups. School B reported a significantly lower liking of the school lunch program than School A (F (1,483) = 8.128, *p* < 0.01) (Figure 1). The school breakfast program was ranked just above the school lunch program for School A and above the disliking of fiber and vegetable groups in School B. The average liking of the school meals did not correlate significantly with the HBIs.

The sleep variables, but not perceived food security, were associated with less healthy HBIs. In School A, students who fell below versus met the recommended sleep hours for their age group trended toward lower average HBIs in ANCOVA, controlling for gender, age, and liking of fun parks, (−2.420 ± 4.633 versus 9.617 ± 3.940) (F (1,148) = 3.769, *p* = 0.054). In School B, greater daytime sleepiness scores were associated with lower HBIs in multiple regression analysis, independent of the influence of covariates (*β* = 0.322, *p* < 0.01). Across both schools, average HBIs did not differ significantly in students who reported food insecurity versus those who did not in ANCOVA, controlling as above (F (1,491) = 1.276, *p* = 0.26). As shown in (Table 3), the PALS groups that were scored into the HBI showed consistent averages across students who reported food insecurity versus not.

### 3.3. Survey Acceptability and Usefulness

Students reported the PALS as acceptable and useful. Across both schools, >85% of students reported agreement (score “agree”<4; whereas 4 =”neither agree nor disagree” and >4 “disagree”) that they could answer the survey questions quickly and without assistance. Somewhat fewer students (73%) agreed that the survey got them to think about what they eat and do. School A versus School B trended toward higher mean responses (2.53 ± 0.087 versus 2.32 ± 0.07, respectively) (F (1,490) = 2.901, *p* = 0.089) for being able to answer the survey questions quickly. Similarly, students in both schools did not demonstrate a significantly in mean difference of their ability to complete the survey without help (F (1,497) = 1.51, *p* = 0.22). However, students in School A reported significantly less agreement that completing the PALS got them to think about their behaviors (3.13 ± 0.11 versus 2.86 ± 0.09, respectively) (F (1,500) = 5.196, *p* = 0.023). 

### 3.4. Tailored Messaging Program

Across both schools, a greater percentage of students received only motivational messages (49.1%) or both motivational and reinforcing messages (41.7%), than only reinforcing messages (8.4%) or no tailored message (i.e., generic message only) (0.8%). Among students in both schools who received at least one motivational message, 30% received messages to substitute a healthier item for a less healthy item, 30% to consume a healthier item, and 40% received both types of motivational messages. Between the schools, these percentages were similar, only differing significantly in the percentage of students who received only reinforcing messages (14% in School A versus 6% in School B) (*x*^2^(2) = 9.14, *p* = 0.01). 

There were no significant differences in the types of messages received by reported food insecurity status (*p* > 0.19), including the percentages of students who received motivational messages, reinforcing messages, or both motivational and reinforcing messages (Table 4). Similarly, no significant differences were found in either measure of insufficient sleep status or percentage of messages received.

### 3.5. Willingness for Behavior Change.

**Figure 3:**
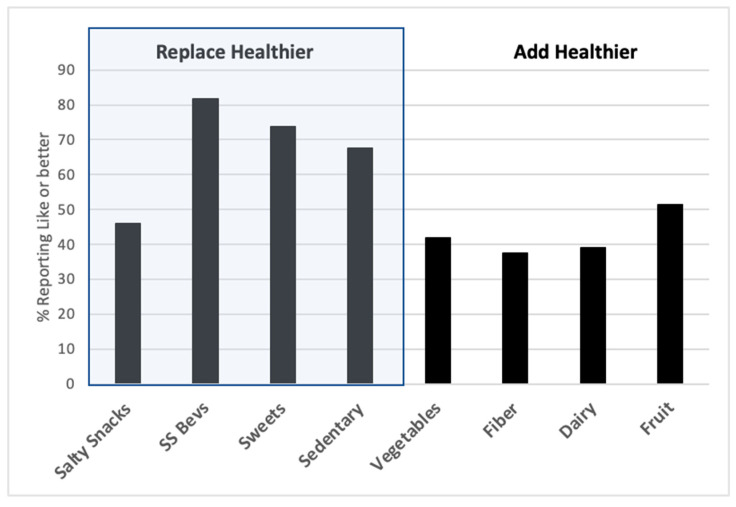
Percentage of students in School A and B who received at least one motivating message reported ≥ “like it” to try the target behavior of the message and more students were willing to replace a less healthy item with a healthier item than to add the healthier item to their diet.

As for the reported liking to address the behavior of the tailored motivational messages, 73% of the students across both schools reported ≥”like it” to try a healthier behavior for at least one of the messages. Judging by message types (as shown in Figure 3), students reported greater level of liking to replace a healthier option for a less healthy option than to add a healthier option, as indicated by the difference in central tendency (43.41 ± 2.74 versus 14.14 ± 2.4, respectively) and distribution towards higher scores (D = 0.3358, *p* < 0.01). The liking ratings of willingness to try the message target behavior did not differ between School A and School B in ANCOVA (*p* > 0.31). By the same token, the willingness to address the target behavior did not vary significantly by food security status or by sleep sufficiency status. 

### 3.6. Message Evaluation

Student ratings of the messages averaged between “agree” and “somewhat agree” across both schools. For their response to the items that measured learning new information from the messages and the messages were helpful, 82.9% and 85.9% (respectively) of students reported agreement (score “agree” <4; whereas 4= ”neither agree nor disagree” and >4 “disagree”). Fewer students, 73.4%, reported wanting to receive more messages in the future. 

Students in School B reported a significantly higher level of agreement to learning new information from the messages than students in School A (F (1,496) =11.22, *p* = 0.01), but no significant differences in the response to the messages being helpful (F (1,496) = 2.108, *p* = 0.15) or wanting more messages in the future (F (1,496) = 1.92, *p* = 0.17). Neither food security nor sleep status influenced the students’ evaluation of the messages.

## 4. Discussions

School-based obesity prevention programs should aim to tailor health promotion efforts to all students as well as provide school-wide strategies to encourage a culture of health, including a healthy school meal program. The present study sought to evaluate the feasibility (acceptability and usefulness) of a school-based, online behavioral screener—from reported liking/disliking of foods and activities (Pediatric-Adapted Liking Survey [8])—with tailored health promotion messages in 505 middle schoolers from two middle schools, one of which was a low-income school (*n* = 310). The PALS plus message program was a collaborative process with the school stakeholders. As outlined in the Assessment phase of an ADAPT-ITT model [26]; (Table 1), interviews with school stakeholders and school meal observations lead to the decision to adapt the PALS plus message program used in a clinical setting [7] to meet the needs of the school while retaining the essence of the original program. Through this collaboration with school stakeholders and topical experts in nutrition, food service, and communication, the PALS was modified based on identified areas for improvement from the School A pilot study [39]. Students completed the program within a single class, with most students (>99%) agreeing to participate. The students could not be identified based on their responses or messages received. In the final testing phase of the modified TTM framework ([26]; Table 1), the data from both schools were analyzed for PALS plus message program acceptability and usability in a school setting. Most students reported ease of completion and that the PALS got them thinking about their dietary and physical activity behaviors. The messages tailored to each student’s PALS responses were well-received, and 73% of students reported willingness to make healthy behavior changes encouraged by the tailored messages. 

The acceptance and usefulness of the PALS and tailored message program did not show differences across the two schools nor did it differ between students who reported concerns with food insecurity (42%) or insufficient sleep (51%). Students reported a greater willingness to improve healthy behaviors by replacing less healthy foods with healthier foods, such as drinking water instead of sugary beverages or eating fruit instead of dessert. They were less willing to add healthier items to their diets, such as adding vegetables or high fiber foods. The reported liking for the school meals averaged close to neutral or disliking. These findings across the schools provided direction for school-wide health promotion efforts.

The PALS was found to be an acceptable and useful method of screening children’s diet and activity behaviors in a school setting, producing similar results to that reported by children (ages 5 to 17 years old) who were recruited with their parents in a clinical setting [7]. Both school and clinical study samples had fun parks as the highest rated item, followed by less healthy foods/beverages and activities, and the healthiest items rated as least liked. Greater preferences were observed for sweet, salty, and fatty foods—as well as lower liking of healthier foods with strong flavors and textures—which is consistent with preference patterns in adolescents that are influenced by genetic and sensory nutrition influences [40]. Strong taste, flavor, and/or texture are key determinants of vegetable preference in children [41] and adults [42] and, in multivariate modeling, social-behavior factors influencing the preference for vegetable, which in turn, influencing the consumption of vegetables [43]. 

The school meal programs can serve as an important vehicle to condition a preference for less preferred, healthier foods in children, especially for the schools in the present study, with meals that follow the U.S. Dietary Guidelines [37]. It is the repeated exposure to foods and tasting foods [44], coupled with positive modeling, nutrition education, cooking experiences [45], storytelling [46], and involvement of the family, that increases an individual’s preference for foods such as vegetables. Attention to student’s food preferences can support improved dietary quality through the school meal program and decrease food waste [47]. The present study did not ask children about their level of exposure to certain foods; therefore, students may be reporting “dislike” to foods they have not tried or only tried a few times. Interventions should focus on increasing consumption of vegetables and other healthy food groups by aiming to improve the school meal program, including repeated exposure to healthy foods, and with parental involvement to provide healthy food choices and to model healthy behaviors at home [48].

School meals are an important source of nutrition, especially for low-income children. Approximately one half (47%) of children and adolescents’ daily energy intake is provided by school meals [49]. These meals in the U.S. needed to change to the 2010–2015 Dietary Guidelines with the Healthy Hunger Free Kids Act in 2010, which increased the quantity and required variety of fruits, vegetables, whole grains, low-fat dairy products with less added sugar, low sodium foods, and foods low in saturated fats since the fall of 2012 [50]. A representative survey of school food service directors showed that elementary student acceptance of the school lunch program improved over the 2012 to 2013 academic year after the changes were implemented [51]. Middle school students showed similar improvements in school meal acceptance [52]. Successful school meal programs are vital to primary obesity prevention through introducing and modeling children to healthy foods and healthy food behaviors. The present findings suggest that, despite school attempts to improve the acceptability of the school meals, more work is needed to make positive changes, through behavioral economics, nudging, and multicomponent programs [53].

The composite index of healthy behaviors (HBI) showed good variability across the sample and acceptable psychometric properties for indexes [54] that are similar to what was previously reported [8]. The HBI also distinguished healthy behaviors between groups previously known to vary in diet and physical activity behaviors. We found that children with greater daytime sleepiness had significantly lower HBIs, which is consistent with findings across a population-based study of Greek children and adolescents [25] as well as a school-based study of adolescents in Brazil [55]. Across the schools, the low-income school (School B) had significantly lower HBIs, tested by central tendency and distribution, than the school with fewer low-income families (School A). The differences in HBIs were driven by lower liking for healthier foods. However, the present study did not find statistically significant average differences in HBIs between students who perceived food insecurity versus those who did not. 

A previous study found lower diet quality among children (3rd to 5th graders) who reported food insecurity, via a study design and multivariate analysis that controlled for parent-reported food assistance and measured body mass index percentile [56]. Our data collection was not able to separate out competing effects on diet quality. Poverty and food insecurity add to many structural determinants of health and influence the ability of students to lead healthy lifestyles. Schools can provide an important function to decrease health disparity and promote health equity through school-based programs, opportunities, policies, and family and community engagement [57]. Interestingly, the students from both schools did not differ in their self-report on how they felt on the day of the survey. Qualitative evaluation of adolescents’ self-ratings found that “feel” is a proxy for health that has more relevance than standard self-rated health questions, especially when the survey is done in a confidential manner [58] through the online survey administered in the present study.

Most students agreed that the PALS was easy to complete, they could complete it without help, and that answering the survey got them to think about their behaviors. These acceptability ratings were similar to those reported by children and their parents in a clinical setting [7]. However, compared to the study conducted in the clinical setting, fewer students in the present study agreed that the survey got them to think about their behaviors (73% of students versus 93% of children in a clinical setting). The context of the school versus healthcare setting and role expectation in a healthcare setting may explain this difference. Completing health surveys can get adolescents to think about their behaviors, draw them to try healthier behaviors based on self-reflection, and encourage them to take ownership of their personal behaviors [59]. School-based follow-up with the survey and empowering the students in health promotion activities can support further self-reflection and action as well as recognizing their individual preferences, especially in a low-income school setting [60].

Behavioral change research has shown that a one-size-fits-all approach is not the most effective strategy and that message tailoring provides relevance for the participants and, therefore, leads to better behavioral change outcomes [61]. In the present study, students received two to three messages tailored to their PALS responses to either motivate or reinforce healthy behaviors. Findings aligned with previous research in a clinical setting, in that significantly more children received motivating messages—as compared to reinforcing messages—and the number of messages and message-type (motivating or reinforcing) did not differ significantly by perceived food insecurity [7]. 

The higher proportion of motivating messages received indicates a higher need for nutrition education and subsequent behavioral interventions in this population. Similar messaging across groups showed that all students were equally likely to receive motivating and reinforcing messages based on their PALS responses and not on their reported characteristics measured in the present study. Across schools, most students reported the messages as helpful (83%) and that they would like to receive more messages in the future (73%). Responses were similar to those of the clinical study, indicating the overall language and message-style to be effective with children in different settings [7]. After receiving the messages and in response to willingness (“like to”) to improve the target behavior, 73% of students reported willingness to change the target behavior—with more students willing to replace a healthier alternative for a less healthy preference (e.g., water for sugary beverage)—than adding a less preferred, healthy item (e.g., adding more whole grains). 

Reported willingness to improve behaviors did not differ significantly by perceived food insecurity or child-reported sleepiness. This suggests that although differences in healthy behavior index scores may be associated with income level, family food insecurity in our previous study [8], or sleepiness status in the present study, children with these characteristics were not targeted with more behavior change messages than those without these characteristics. This finding agrees with previous research [7] and aligns with the TTM [62] that the PALS and tailored messaging program may promote behavior change in children. Similar findings in message acceptance and usefulness, along with the existing literature on the efficacy of technology use coupled with tailored feedback [63], show that a more individualized tailored messaging program could be a useful tool for promoting behavioral change in children in both a clinical and school setting. 

Tailored messaging program in the form of mHealth or eHealth may be the most effective strategy in reaching adolescents. Previous research suggests that daily text messaging may be effective in motivating behavioral change [64]. A systematic review showed the effectiveness of this method of tailored messaging through eHealth in the form of text messaging, email, or online websites [65], which allows for more anonymity among participants. Most studies included in the systematic review also found the most success in interventions or messaging that provided specific goals and clear feedback to participants, instead of delivering general health information [65]. The present study showed acceptance of the use of an online modality to provide tailored messaging with students reporting the desire to receive more messages in the future; however, messages were only provided at one instance in time. Continued health promotion efforts may benefit from incorporating regular follow-up messaging, as suggested by other research studies [64]. This regular messaging may also be successful in collaboration with school-based activities and interventions [65].

School-wide information from the PALS with message program could provide potential new directions for school-wide health program messages. Repeated exposure to and visibility of health messages has been shown to drive healthy behavior change in middle schoolers in interventions that focused on nutrition and physical activity [66]. The same campaign also found success in constantly changing campaign messaging to maintain the interest of the students [66]. Other studies found similar success in health campaigns that incorporated student-generated messages to promote student engagement and acceptance by peers [67]. In the present study, School A students were involved in the development of school-wide messages on reducing food waste in the cafeteria and breakfast and voting on the message that they liked best, based on the findings generated in the current PALS that was integrated with a tailored message program [39]. Students who assisted in message-development also acted as the peer influencers to help the research team drive the intervention [67]. 

Other studies incorporated social media platforms such as Instagram and Twitter to spread health information and connect with more students. The use of social media also allowed for more student engagement with their ability to interact with and share health communication postings [68]. Overall, school-wide health promotion campaigns are most effective when using messaging familiar to and well-accepted by students with repeated exposure. The present study provides the tools to build an effective school-wide messaging campaign—through tailored messaging—and student-reported message usefulness and acceptance of the messages. In addition, the universal disliking for vegetables and whole grains, and the low interest in adding healthy foods are findings that could help guide an intervention approach and school-wide campaigns—to focus on these food items and strategies to connect with community-based programs—such as involving students in designing healthy and acceptable school menus and putting fruits/vegetables harvested from community and school-based gardens on the menu.

### Strengths and Limitations

The present study has strengths and limitations to note. The study strengths include the generalizability due to the use of similar surveys in two schools with different distributions of age, race/ethnicity, family food insecurity, and sleep status of its students. The PALS was previously validated for use in a clinical setting to assess children’s diets and physical activities that also tested tailored health messages [8]. Another strength is that the PALS can be quickly completed during a single class session, reducing participant burden and has been reported as easy to use, acceptable, and useful by previous [7] and current participants. The use of online survey technology is a study strength because it allows for tailored messages to be delivered to many students in an instant, allowing for the ability to reach more students at once, including those enrolled in hybrid or distance-learning programs. Technology-based surveys have also been previously found to provide more accurate responses than traditional paper-and-pencil surveys, proving a more accurate representation of diet quality across schools [31]. Child-reported or perceived food insecurity questions were extracted from the validated measure of food insecurity status [69]. 

The present study also had limitations. One is that the impact of the PALS plus message program on actual behaviors was not measured. However, in a pre-posttest pilot study within School A, the PALS responses became the basis of a school-wide, low-impact nutrition education and message program that reinforced the school meal program, with repeat PALS plus message program at the end of the school year. Preliminary findings across the school supported that PALS plus message program and school-wide effort could motivate healthier diet and physical activity behaviors [39]. This effort was prevented in School B due to the COVID-19 pandemic. Another limitation of the present study was that we analyzed the data by broad message category (motivational versus reinforcing) and not by single message type. The algorithms embedded in the survey technology were not set up to allow for analysis by specific message type because of the need to limit the total number of messages each child received. A third limitation of the present study is that behaviors were not assessed at home. The literature shows that adolescents tend to consume energy-dense snacks containing few micronutrients when eating at home or independently [70]. Thus, behaviors assessed at school may only reflect children’s most healthy dietary habits—compared to those at home—due to the balanced school meals provided at school. Future research using the PALS may benefit from asking participants about behaviors practiced at home in addition to behaviors at school. Future studies would benefit from following a pre-/post-survey, controlled design with follow up messages sent to participants. This would allow future researchers to track behavior change among participants over time. Another limitation is the possibility of response bias—students may not have answered all survey questions honestly or may have rushed through the questions. To combat this, students were asked to take their time, answer the survey questions honestly, and were assured that their responses would remain anonymous and confidential. Furthermore, the acceptability and usefulness of the PALS was not tested among school faculty and community stakeholders. Although community stakeholders responded positively to the PALS, future research may benefit from a formal evaluation to allow for anonymity and more detail in responses. 

## 5. Conclusions

The PALS and tailored messaging program were found to be acceptable and useful in assessing diet quality and physical activity among students from two middle schools, one of which had a higher percentage of impoverished families. Students’ responses to the PALS were similar across schools, showing generalizability across adolescents of different ages, race/ethnicity, income-level, food insecurity status, and sleep sufficiency. The PALS can be used to drive school-wide efforts of health promotion and obesity prevention. However, it is unknown if the PALS and tailored messaging program alone could lead to true behavior change because the present study was conducted in a single session at each school and behavior change was not assessed at follow up due to the COVID-19 pandemic. It is likely that the repeat PALS and tailored messaging program coupled with parental involvement and school-based programs could lead to true behavior change among children, based on child-reported willingness to improve target healthy behaviors. The use of this online survey technology with tailored messaging may benefit future health promotion programs, as it has shown acceptability, and usefulness in both clinical [7] and school-based settings.

## Figures and Tables

**Table 1:** Applying the modified ADAPT-ITT Model [26] to the Pediatric Adapted Liking Survey (PALS) plus tailored messages program for students in school A and school B.

Phase	Methodology
1. Assessment	Conducted interviews with community stakeholders to identify concerns with children’s health behaviors and food security. Conducted school meal observations and plate waste studies to assess children’s response to the school nutrition programs.
2. Decision	Decision to use validated behavioral screening [8] (PALS survey) and tailored message program from a clinical setting [7] to a school-setting. Decision to adapt the program as the evidence-based intervention to address the perceived needs of the school.
3. Administration	Collaboration between the research team and stakeholders in the school and community to adapt the program to School A.
4. Production	Produced the adapted program and conducted with students in School A. Produced reports for stakeholders regarding program findings. Identified areas for improvement while maintaining the theory-based core elements of the program and for comparability across schools.
5. Topical Experts	Identified nutrition, school foodservice, and communications experts. Identified another school to test the adapted program and test the generalizability of findings. Added the improvements with School B stakeholder feedback on the program administration, including functional outcomes of insufficient sleep.
6. Integration	Conducted the program in School B maintaining key components to allow integration of findings across both schools.
7. Testing	Analyzed results across School A and B to determine acceptability and usefulness of the adapted program.

**Table 2:** Description of students by middle school.

		% of Participants: School A (*N* = 195)	% of Participants: School B (*N* = 310)
Age	Ten Eleven Twelve Thirteen Fourteen Other	0 10.5 80.0 8.5 1.0 0	1.3 34.8 34.2 24.2 3.9 1.3
Gender	Male Female Other	51 49 N/A	48 49 3
Race/Ethnicity	White Black/African Am. Hispanic/Latino Asian American Indian Other Pacific Islander Multiple Declines to Answer/ Don’t know/Not sure	22.5 34.0 10.0 2.0 0.5 0.5 21.5 6.0 3.0	9.7 21.0 40.6 9.0 0.3 0 12.9 2.6 3.9
How do you feel today?	Smile rating Neutral Frown rating	68 22 10	68 24 9
Food Insecurity †	Food Secure Food Insecure	61 39	56 44
Sleep ‡	Insufficient Sleep Sufficient Sleep	43 57	55 45

† Reporting “sometimes” or “often” to one of three questions about food insecurity. ‡ Insufficient sleep was defined differently across schools—School A was less than the recommended sleep hours per age group (<10 hours for 10–11 years; <8.5 hours for 12–14 years) [27]; School B was ≥15 on the summed score across eight items of Pediatric Daytime Sleepiness Scale [24].

**Table 3:** PALS food and activity groups and Healthy Behavior Index (HBI) scores (and its diet and physical activity groups) in middle school students (School A and School B) by self-reported food security versus food insecurity †.

	Food Secure *N* = 283	Food Insecure *N* = 209
Sedentary	63.77 ± 1.65	64.69 ± 1.92
Sweet	55.94 ± 1.85	59.55 ± 2.15
Phys Act	50.47 ± 2.05	40.70 ± 2.38
Sugar Sweetened Beverages	47.96 ± 2.14	49.29 ± 2.49
Salty	43.10 ± 1.93	46.66 ± 2.23
Fruit	37.56 ± 2.27	34.42 ± 2.64
Dairy	27.16 ± 2.40	27.27 ± 2.79
Protein	19.14 ± 1.93	17.47 ± 2.24
Fiber	2.00 ± 2.31	4.10 ± 2.68
Vegetable	−9.11 ± 2.90	−12.3 ± 3.32
HBI ‡	−5.16 ± 2.19	−9.00 ± 2.55

† Students who responded “sometimes” or “often” to at least one of the three questions about food insecurity were considered to be food insecure. Only students that responded “never” to all three questions were considered to be food secure. ‡ F (1,491) = 1.276, *p* = 0.26.

**Table 4:** Percentage of food secure and food insecure students (by self-report) within each school by health promotion message type †. Chi-squared testing within a school was non-significant.

	*School A*		*School B*	
	Food Secure	Food Insecure	Food Secure	Food Insecure
Only Reinforcing	11.8	14.9	5.8	5.8
Only Motivating	47.4	45.6	56.9	46.8
Both Types of Messages	40.8	39.5	37.3	47.4

† Message wording is similar to the previously published [7].

## Data Availability

The data presented in this study are not available as it was not part of the Internal Review Board approval.
